# Cholecalciferol Additively Reduces Serum Parathyroid Hormone and Increases Vitamin D and Cathelicidin Levels in Paricalcitol-Treated Secondary Hyperparathyroid Hemodialysis Patients

**DOI:** 10.3390/nu8110708

**Published:** 2016-11-05

**Authors:** Jing-Quan Zheng, Yi-Chou Hou, Cai-Mei Zheng, Chien-Lin Lu, Wen-Chih Liu, Chia-Chao Wu, Ming-Te Huang, Yuh-Feng Lin, Kuo-Cheng Lu

**Affiliations:** 1 Division of Critical Care Medicine, Department of Emergency Medicine-Critical Care Medicine (EM-CCM), Shuang Ho Hospital, Taipei Medical University, New Taipei City 23561, Taiwan; 16044@s.tmu.edu.tw (J.-Q.Z.); thanthanwinnge@gmail.com (M.-T.H.); 2 Graduate Institute of Clinical Medicine, College of Medicine, Taipei Medical University, Taipei 11031, Taiwan; 11044@s.tmu.edu.tw (C.-M.Z.); janlin0123@gmail.com (C.-L.L.); wayneliu55@gmail.com (W.-C.L.); linyf@s.tmu.edu.tw (Y.-F.L.); 3 Division of Nephrology, Department of Internal Medicine, Cardinal Tien Hospital, School of Medicine, Fu-Jen Catholic University, New Taipei City 23148, Taiwan; athletics910@gmail.com; 4 Department of Internal Medicine, School of Medicine, College of Medicine, Taipei Medical University, Taipei 11031, Taiwan; 5 Division of Nephrology, Department of Internal Medicine, Shuang Ho Hospital, Taipei Medical University, New Taipei City 23561, Taiwan; 6 Division of Nephrology, Department of Medicine, Tri-Service General Hospital, National Defense Medical Center, Taipei 11490, Taiwan; wucc@ndmctsgh.edu.tw; 7 Division of General Surgery, Department of Surgery, Shuang Ho Hospital, Taipei Medical University, New Taipei City 23561, Taiwan

**Keywords:** maintenance hemodialysis (HD), secondary hyperparathyroidism (SHPT), paricalcitol, cholecalciferol, study, human cathelicidin (hCAP-18)

## Abstract

Background: Active Vitamin D analogues are used clinically for prevention and treatment of secondary hyperparathyroidism (SHPT) in hemodialysis (HD) patients. Nutritional vitamin D supplementation is used for additional local parathyroid (PTH) suppression, with lower incidence of hypercalcemia and hyperphosphatemia. This study evaluates the possible beneficial effects of combined vitamin D treatment (paricalcitol and cholecalciferol). Methods: Sixty HD patients with serum parathyroid hormone (iPTH) >300 pg/mL were enrolled. All patients administered 2 mcg/day of paricalcitol and were randomly allocated into control group (placebo) or study group (cholecalciferol) for 16 weeks. Serum 25(OH)D_3_, iPTH and human cathelicidin (hCAP-18) were measured at baseline and during follow-up. Results: iPTH levels decreased in the study group appropriately and were more significantly decreased at 16 weeks. Study group had significantly increased 25(OH)D_3_ levels. In addition, the study group had significantly increased serum hCAP-18 levels compared with control group. Correlation analysis showed a significant correlation between the percentage increase in serum hCAP-18 and 25(OH)D_3_ levels. Conclusions: Cholecalciferol, in combination with paricalcitol, additively lowers the iPTH levels in a significant number of patients after 16 weeks of supplementation. A dose of 5000 IU/week of cholecalciferol could maintain serum 25(OH)D_3_ levels above 30 ng/dL as early as 8 weeks after beginning supplementation. Doubling of serum cathelicidin levels were noted after 16 weeks of cholecalciferol supplementation in 40% of study patients.

## 1. Background

Vitamin D naturally acquired from the diet or dermal synthesis (ergocalciferol, cholecalciferol) is converted into more active vitamin D compounds with the help of liver 25α-hydroxylase (25α-OHase) for 25-hydroxyvitamin D (calcidiol, 25(OH)D), and kidney 1α-hydroxylase (1α-OHase; CYP27B1) for 1,25-dihydroxyvitamin D (1,25(OH)_2_D), respectively. The enzyme 24-hydroxylase (24-OHase) is responsible for degradation of the vitamin D analogs 25-hydroxyvitamin D (calcidiol, 25(OH)D) and 1,25-dihydroxyvitamin D (1,25(OH)_2_D). With progressive kidney function impairment, end stage kidney disease (ESKD) patients experience a decrease in 1α-OHase activity and in 1,25(OH)_2_D production, with subsequent hypocalcemia and secondary hyperparathyroidism (SHPT). Active vitamin D analogs (e.g., calcitriol, paricalcitol) are used clinically to prevent or treat the SHPT and mineral disorders among ESKD and dialysis patients. However, longer use of active vitamin D analogs revealed hypercalcemia, vascular calcification [1] and cardiovascular adverse effects [2] which flawed the therapeutic goals.

Circulating parathyroid (PTH) levels have been found to be inversely correlated with serum 25(OH)D_3_, a circulating vitamin D metabolite which indicates vitamin D status [3,4,5,6,7]. With the help of local 1α-OHase activity, 25(OH)D_3_ serves as an important substrate for the local generation of 1,25(OH)_2_D [8,9]. Most ESKD patients have vitamin D deficiency, which is indicated by low serum 25(OH)D_3_ levels [10,11,12,13]. Segersten et al. [14] revealed 1α-OHase expression in parathyroid glands which presumably suppressed the PTH gland hyperplasia in an autocrine/paracrine manner. A previous study revealed coincident increased expression of 1α-hydroxylase (increased by approximately 10-fold) and reduced 24-hydroxylase in most SHPT glands [15], and highlighted the requirement for more 25(OH)D_3_ in these patients. Ritter et al. [16] demonstrated that the local effect of 25(OH)D_3_ in PTH suppression possibly occurs through direct activation of vitamin D receptor (VDR) in parathyroid glands. Furthermore, 25(OH)D_3_ played less of a role in hypercalcemia and mineral disturbances. These findings explain the possible additive role of nutritional vitamin D (cholecalciferol) supplementation to active vitamin D analogs in SHPT patients.

Similarly, the non-classical actions of vitamin D take place by local active vitamin D in extra-renal tissues (e.g., immune cells) through local 1α-hydroxylation. Deficient 25(OH)D_3_ has been associated with inadequate non-classical effects despite higher extrarenal 1α-hydroxylation status among chronic kidney disease (CKD) patients [17,18]. Human cathelicidin (hCAP-18) is an antimicrobial peptide that is transcriptionally regulated by active 1,25(OH)_2_D [19,20] and has pleiotropic immunomodulatory effects. Locally active 1,25(OH)_2_D in immune cells is responsible for the production of human cathelicidin (hCAP-18) and enhances the capacity of autophagy through toll-like receptor activation [21,22,23]. A previous study revealed that chronic hemodialysis (HD) patients with low baseline plasma human cathelicidin (hCAP-18) levels had an independently associated higher risk of infection-related mortality [24]. However, the effects of nutritional vitamin D (cholecalciferol) supplementation on serum hCAP-18 levels in dialysis patients are still unknown.

In our recent study, we hypothesized that cholecalciferol supplementation generates additive SHPT control with active vitamin D analogs (paricalcitol) and increases 25(OH)D_3_ and hCAP-18 levels effectively in regular HD patients.

## 2. Methods

### 2.1. Study Design

This was a prospective, randomized, interventional, efficacy study comparing cholecalciferol plus paricalcitol with a standard vitamin D therapy, paricalcitol plus placebo (control group). Patients were given an oral form of cholecalciferol 5000 IU per capsule (Healthy Origens, Pittsburgh, PA 15241, USA) in the study group and cholecalciferol placebo (olive oil) was used in the control group. A baseline visit was performed just before the start of the study, and further study visits were performed at 4, 8, 12 and 16 weeks after baseline. Both patients and/or investigators were blinded to the study intervention. Prior to the study, G*power was used to calculate the required sample size [25] and effects were detected in a two-sided test with a power of (1 − β) = 80% at a significance level of 0.05. Other calculation settings were as follows: (1) the randomization process was based on 1:1 proportion of this study; (2) the effect size was set as 0.8 because we considered the cholecalciferol intervention as having a large effect. Based on these settings, the required sample size for calculating was at least 25 subjects in the cholecalciferol group and 25 subjects in the placebo group. We used 30 subjects in each study group.

### 2.2. Study Approval and Informed Consent

The trial was conducted in Taipei Medical University Shuang Ho Hospital from 1 June 2014 to 31 May 2015. The study was approved by the authorities of the Institutional Review Board of Taipei Medical University, and all the patients provided written consent before study enrollment.

### 2.3. Patient Eligibility and Randomization

**Figure 1:**
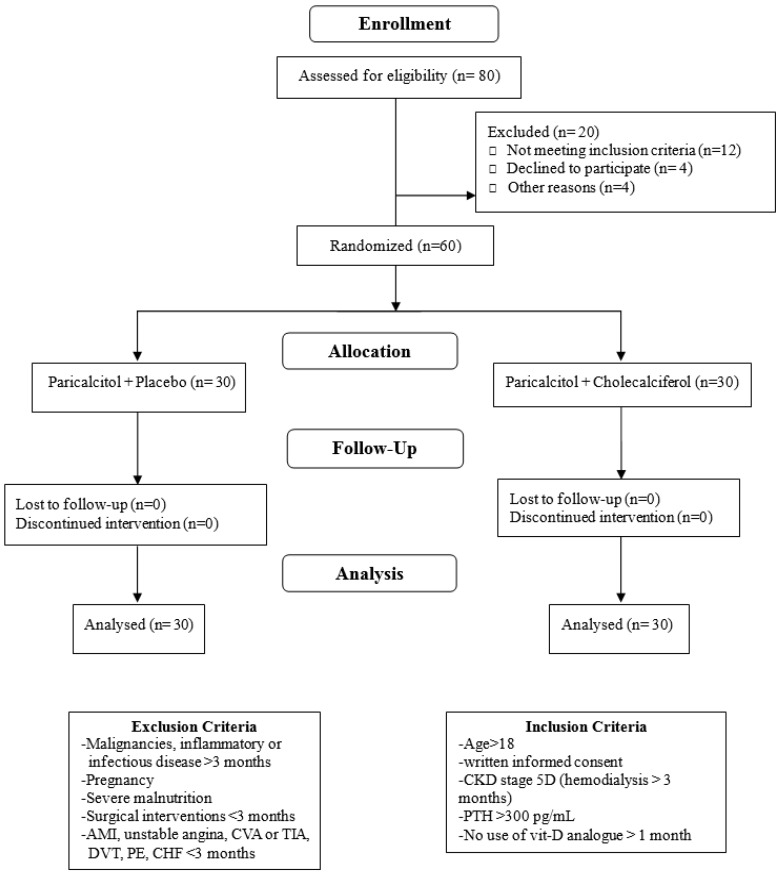
Study participants’ flow chart. AMI: acute myocardial infarction; CVA: cerebrovascular accident; TIA: transient ischemic attack; DVT: deep vein thrombosis; PE: pulmonary embolism; CHF: congestive heart failure; CKD: chronic kidney disease; PTH: parathyroid hormone.

Eligible patients were 18 years or older, on HD therapy for at least three months with concomitant SHPT (serum parathyroid hormone (iPTH) > 300 pg/mL). Dialysate Ca was 1.5 mmol/L, and the patients had not been using any form of vitamin D analogs for at least one month before the start of the study. Patients under regular treatment with calcitriol (*n* = 29) had this medication withdrawn one month before the start of the study. Only non-calcium containing phosphate binders were administered. The patients were excluded if they were pregnant, breastfeeding, or of childbearing potential and not practicing birth control. Those with malignancies, severe malnutrition, and inflammatory or infectious disorders diagnosed more than three months before the start of the study were also excluded. Other exclusion criteria included surgical interventions and vascular diseases, including acute coronary syndrome, unstable angina, cerebrovascular accident, transient ischemic attack, deep vein thrombosis, pulmonary embolism, or congestive heart failure within three months of the study period. The patients were also excluded if they had a history of allergy to cholecalciferol or if the investigator considered one-month withdrawal of paricalcitol unsuitable (Figure 1).

### 2.4. Treatment Intervention

All the patients administered 2 mcg of paricalcitol (19-nor-α-25-dihydroxy ergocalciferol) daily (fixed dose protocol) and were randomly allocated into two groups with placebo or with low-dose cholecalciferol supplementation (5000 IU/week). Follow-up visits were scheduled at week 4, 8, 12 and 16 after the start of the medication. Serum concentrations of 25(OH)D_3_, iPTH and hCAP-18 were assessed at baseline and during follow-up.

Serum 25(OH)D was determined by ELISA according to the manufacturer’s instructions (Immundiagnostik AG, Bensheim, Germany). The serum samples were measured in triplicate. The coefficients of variation for 25(OH)D measurements were <3% at levels <30 ng/mL. Serum iPTH was measured using a kit from Immutopics Inc., San Clemente, CA, USA. The PTH kit had a detection limit of 13 pg/mL, with intra-assay Coefficient of Variability (CV) of 4.5% and inter-assay CV of 4.8%. Plasma cathelicidin (hCAP-18/LL-37) was measured by ELISA (Hycult Biotech, Frontstraat 2a 5405 PB Uden, The Netherlands). hCAP-18 measurements were conducted in duplicate after initial thaw of the serum samples, and the calculated mean of the measurements was used in the analyses. The intra-assay CV for hCAP-18 measurements was <10%. Serum levels of Ca, P, and alkaline phosphatase (ALP) were also monitored. Hospital records were obtained and examined by two practicing nephrologists (Figure 1).

### 2.5. Objectives, Outcomes and Measures

The primary study objective was to assess the beneficial effects of cholecalciferol combined with paricalcitol in HD patients with concomitant SHPT.

Primary Outcome: The primary therapeutic outcome was serum iPTH levels less than 300 pg/mL at the end of the study.

Secondary Outcome: The secondary outcomes were serum 25(OH)D**_3_** more than 30 ng/mL and doubling of serum cathelicidin (hCAP-18) level at the end of the study.

### 2.6. Data Collection and Statistical Analysis

According to data distribution, the results were expressed as mean ± standard deviation or median (interquartile range). Parametric or nonparametric tests were used for analysis; for paired data, the Student *t* or Wilcoxon tests, respectively, and for between-group comparisons, the Student *t*, one-way ANOVA or Mann–Whitney U tests were used. Unilateral correlation analysis was performed using Pearson (*r*) or Spearman correlation (r_s_), as appropriate. All the tests were two-sided, and *p* < 0.05 was considered statistically significant. Statistica (Version 11, Stat Soft, Inc., 2300 East 14th Street Tulsa, OK 74104, USA) was used for calculations.

## 3. Results

### 3.1. Patient Recruitment and Analysis Sets

Table 1 shows the demographic characteristics of the 60 patients. Patients were well matched by treatment allocation. All the patients experienced CKD stage 5D with SHPT with 16 men (53.3%); mean age, 65.2 ± 12.4 years in the paricalcitol plus placebo (control group) versus (vs.) 14 men (46.7%); mean age, 64.8 ± 13.2 years in the paricalcitol plus cholecalciferol (study group). Dialysis vintage and body mass index (BMI) were not significantly different between two groups. The causes of ESKD were diabetes mellitus (*n* = 31), primary glomerulonephritis (*n* = 5), hypertension (*n* = 10), and others (e.g., vascular or ischemic nephropathy, tubulointerstitial nephritis, etc.) (*n* = 16). No significant difference was noted regarding the prior calcitriol usage (*n* = 8 in control group vs. *n* = 10 in study group). All patients had high serum iPTH levels (control group, 677.5 ± 167.8 pg/mL vs. study group, 689.0 ± 180.1 pg/mL) and low 25(OH)D_3_ levels (control group, 19.53 ± 8.2 ng/mL vs. study group, 19.6 ± 7.3 ng/mL). Serum albumin, ALP, albumin-corrected Ca, P, and hCAP-18 levels were not significantly different between two groups. Table 2 shows the serum biochemistry parameter changes after 16 weeks of treatment.

### 3.2. Changes in Serum PTH Levels within and between Groups during Study Period

**Figure 2:**
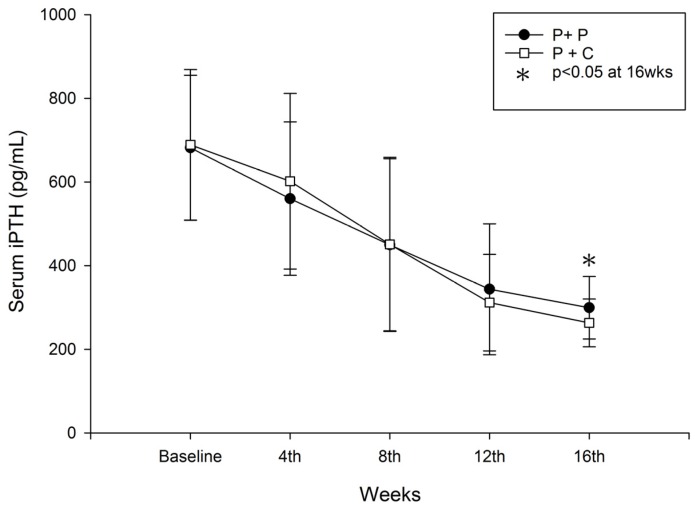
Changes in serum iPTH during the study period in both groups (P + P: paricalcitol plus placebo; P + C: paricalcitol plus cholecalciferol, * *p* < 0.05: P + P vs. P + C).

Serum iPTH most significantly decreased at 16 weeks in the study group, from 689.0 ± 180.08 pg/mL to 262.9 ± 57.14 pg/mL in the study group (*p* < 0.05) vs. from 681.9 ± 173.3 pg/mL to 299.37 ± 74.62 pg/mL in the control group (*p* < 0.05) (Figure 2). The lowering of iPTH level was not significantly different in both groups at weeks 4, 8 and 12 weeks. From between group analysis, we found that 6.7%, 23%, 40% and 50% of the control group; and 6.7%, 23%, 60% and 76.7% of the study group achieved target iPTH level at weeks 4, 8, 12 and 16 respectively. Significantly more patients in study group achieved target level at week 16 of the study (15/30 vs. 23/30, *p* = 0.00032 (Chi-Square test)) (Table 3).

### 3.3. Changes in Serum 25(OH)D_3_ Levels within and between Groups during Study Period

**Figure 3:**
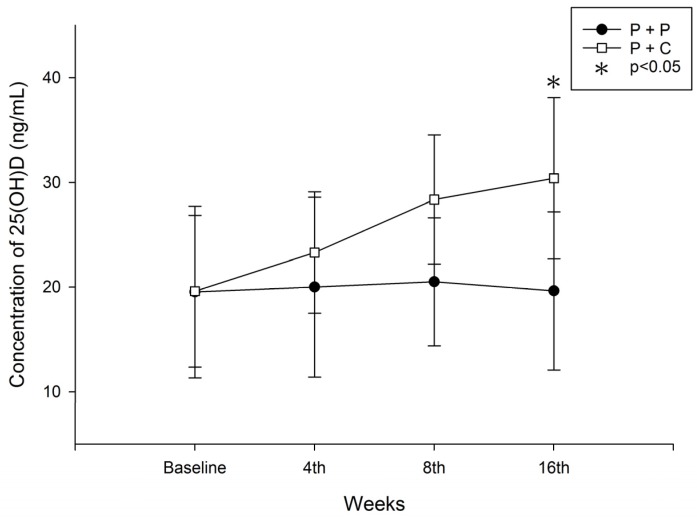
Changes in **s**erum 25(OH)D_3_ levels during study period (P + P: paricalcitol plus placebo; P + C: paricalcitol plus cholecalciferol, * *p* < 0.05; P + P vs. P + C).

In the study group, 25(OH)D_3_ levels increased from 19.6 ± 7.26 ng/mL to 30.4 ± 7.7 ng/mL (*p* < 0.05). However, in the control group, 25(OH)D_3_ levels did not change significantly (from 19.53 ± 8.2 ng/mL to 19.63 ± 7.56 ng/mL) (Figure 3). Between group analysis found that 20%, 6.7%, 13.3% and 10% in the control group; and 13.3%, 50%, 53.3% and 60% of study patients achieved target vitamin D levels by weeks 4, 8, 12 and 16 respectively. The number of patients achieving our secondary outcome (25(OH)D_3_ ≥ 30 ng/dL) was significantly higher in the study group from the 8th week of the study onwards (2/30 vs. 15/30, *p* = 0.001 at 8th week; 4/30 vs. 16/30, *p* = 0.001 at 12th week and 3/30 vs. 18/30, *p* = 0.001 at 16th week respectively (Chi-Square test)) (Table 3).

### 3.4. Changes in Serum Cathelicidin (hCAP-18) Levels within and between Groups during the Study Period

**Figure 4:**
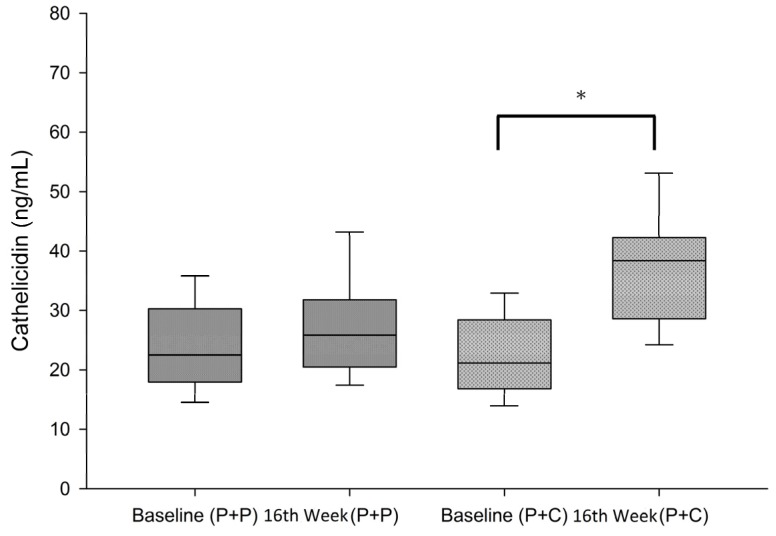
Changes in serum cathelicidin (hCAP-18) levels during study period (P + P: paricalcitol plus placebo, P + C: paricalcitol plus cholecalciferol; * *t*-test, Baseline vs. 16th week in P + C group, *p* < 0.05).

Study group had significantly increased serum hCAP-18 levels from baseline compared with control group (from 22.25 ± 6.71 ng/mL to 82.13 ± 68.67 ng/mL in the study group (*p* < 0.05) vs. from 24.05 ± 7.99 ng/mL to 26.59 ± 65.68 ng/mL in control group) (Figure 4). Between group analysis revealed 40% of the study group as compared with only 6.7% of control group achieved the doubling of hCAP-18 level at week 16 of the study (2/30 vs. 12/30, *p* = 0.006 (Chi-Square test)) (Table 3).

### 3.5. Correlation between the Change in Serum 25(OH)D_3_ and hCAP-18 Levels after 16 Weeks of Study in HD Patients with SHPT

**Figure 5:**
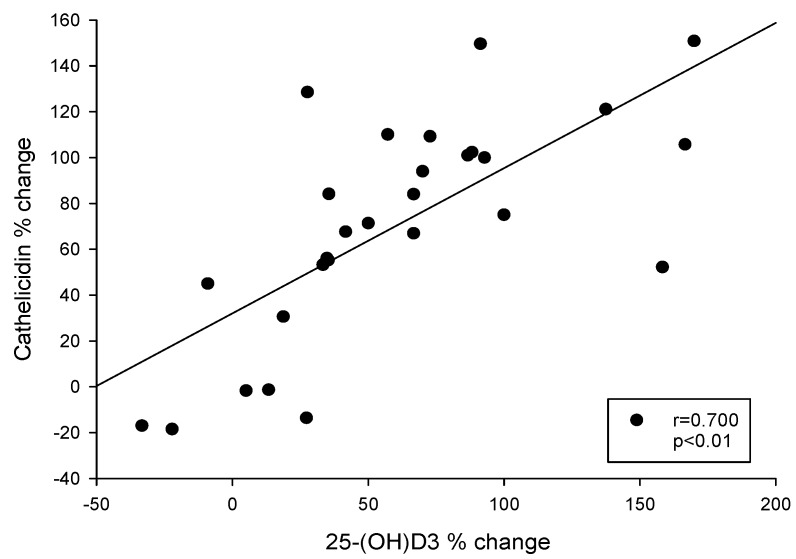
Correlation between percentage change of serum cathelicidin and 25(OH)D_3_ between baseline and 16 weeks after initiation of dual vitamin-D (paricalcitol + cholecalciferol) treatment.

Correlation analysis revealed a significant correlation between the percentage increase (from baseline to 16 weeks in the study group) in serum hCAP-18 and 25(OH)D_3_ levels (*p* < 0.05) (Figure 5).

## 4. Discussion

Our primary and secondary outcomes were met at the end of the study period in the cholecalciferol supplementation group. Additive PTH lowering was found in a significant number of study patients after 16 weeks of supplementation. A dose of 5000 IU/week of cholecalciferol could maintain serum 25(OH)D_3_ levels above 30 ng/dL as early as 8 weeks after start of supplementation. Doubling of serum cathelicidin levels was noted with 16 weeks of cholecalciferol supplementation in 40% of study patients. 

Active vitamin D compounds were traditionally used as sole agents among SHPT patients for its inhibition effects on PTH gene transcription and chief cell hyperplasia. Growing evidence shows that higher doses of active 1,25(OH)_2_D may aggravate 25(OH)D_3_ deficiency because of its feedback inhibition of hepatic 1α-OHase and 25α-OHase, which results in 25(OH)D_3_ shortage among other tissues or organs (e.g., colon, osteoblasts, parathyroid gland, monocytes, and immune cells, etc.) [26,27]. Active 1,25(OH)_2_D also induces the 24-OHase enzyme and autoregulates its own catabolism [28]. Thus, active 1,25(OH)_2_D may not only reduce 25(OH)D_3_ production but also increase 1,25(OH)_2_D and 25(OH)D_3_ catabolism. Clinically, serum 25(OH)D_3_ levels decline since early CKD and low serum 25(OH)D_3_ levels might increase serum iPTH levels, suggesting that this event is crucial for SHPT development. Several factors, including aging and comorbidities such as diabetes and hypertension, were consistently associated with low 25(OH)D_3_ levels in both dialysis and nondialysis CKD [29]. A previous study of predialysis CKD patients also revealed that diabetes and obesity are risk factors for 25(OH)D_3_ deficiency [29]. In addition, 25(OH)D_3_ levels higher than 20 ng/mL seem sufficient to control serum PTH in patients with CKD [30]. Studies among dialysis patients are rare, and although our cohort included a with relatively small number of patients, all were under regular dialysis with moderate to severe SHPT.

We found that our primary outcome, a iPTH level less than 300 pg/mL was achieved in both control and study group at the end of study, with significantly lower PTH level at week 16 in the cholecalciferol supplementation group (Figure 1). Furthermore, 76.7% of study patients achieved target iPTH level (iPTH ≤ 300 pg/mL) compared to 50% of the control group (Table 3). Circulating 25(OH)D_3_ is important for non-classical actions of vitamin D in extra-renal tissues by converting to active 1,25(OH)_2_D through local 1α-OHase activity. Furthermore, circulating 25(OH)D_3_ levels also increase 1α-OHase activity in a substrate-dependent manner among CKD patients; a higher concentration of 25(OH)D_3_ is associated with stronger 1α-OHase activity and vice versa [12]. Recent studies also showed the importance of circulating 25(OH)D_3_ in autoregulation of the PTH glands and probably through direct activation of the vitamin D receptor (VDR) within parathyroid cells [14,16]. Another study also revealed increased expression of 1α-hydroxylase (approximately increased in 10 folds) and reduced 24-hydroxylase in most SHPT glands [15] which further highlighted a greater 25(OH)D_3_ requirement among these patients. Although 25(OH)D_3_ had several hundred times lower VDR binding affinity and lower suppressing PTH activity than 1,25(OH)_2_D_3_ [16], circulating 25(OH)D_3_ levels were a thousand times higher than 1,25(OH)_2_D levels. These findings explain the critical need for 25(OH)D_3_ supplementation in SHPT patients in addition to active vitamin D analogs. Indeed, our study results revealed that additional reduction in PTH levels was achieved in almost 76.7% of the study patients with cholecalciferol supplementation.

In accordance with previous studies, we found that 25(OH)D_3_ levels significantly increased in the study group, whereas they remained low in the control group (Figure 3). The number of patients who achieved the target level was significantly increased in the study group from the 8th week of supplementation (Table 3). Clinical studies of dialysis patients with SHPT also reported that the combined use of nutritional vitamin D might reduce the active vitamin D doses required for SHPT control and related adverse effects [2,31,32]. However, studies on the effective dosage of vitamin D supplementation for SHPT remain controversial [33,34,35]. In our study, we used a low dose of cholecalciferol (5000 IU/week) and found that it could effectively increase the 25(OH)D_3_ levels. Sixty percent of study patients achieved target level (25(OH)D_3_ ≥ 30 ng/dL) with cholecalciferol supplementation for 16 weeks (Table 3). This was consistent with a study conducted by Dusilova-Sulkova et al. [36]. By contrast, Dogan et al. reported that a 300,000 IU bolus of cholecalciferol significantly reduced PTH in patients with CKD stage 3–4 with a one-month follow-up period [37]. The Dialysis Infection and Vitamin D in New England (DIVINE) study [38] compared the effects of 50,000 IU of oral ergocalciferol weekly, 50,000 IU of oral ergocalciferol monthly, or placebo in maintenance dialysis patients. The study found that sufficient vitamin D levels were achieved in 91% with weekly dosing, and in 66% with monthly dosing, compared to 35% with placebo at 12 weeks of treatment.

The next major observation of our study was that cholecalciferol supplementation effectively increased the serum cathelicidin levels, in contrast to the control group (Table 3). Doubling of serum cathelicidin levels was noted in up to 40% of those with cholecalciferol supplementation. The percentage increase (from baseline to 16 weeks of dual vitamin D treatment) in serum cathelicidin and 25(OH)D_3_ levels were found to closely correlated in correlation analysis (*p* < 0.05) (Figure 5). Cathelicidin is a crucial antimicrobial peptide in innate immunity regulated by vitamin D [39]. It’s well known that low plasma level of cathelicidin predicts increased infectious disease mortality in patients undergoing hemodialysis [40]. Leaf et al. revealed that lower serum cathelicidin and 25(OH)D_3_ levels predict higher in-hospital mortality [41]. Many in vitro and in vivo studies have reported the antimicrobial effect of vitamin D through the increased production of cathelicidin [42,43,44,45]. Bacchetta et al. revealed that vitamin D supplementation in vitro and in vivo promotes innate immune responses that may enhance macrophage antibacterial responses in patients undergoing peritoneal dialysis [46]. A high dose of oral cholecalciferol modulates disease-relevant T-cell functions and increases serum cathelicidin in patients with Human immunodeficiency virus (HIV) infection [47]. In critically ill patients with severe sepsis, high-dose cholecalciferol elevates serum cathelicidin with serum 25(OH)D_3_ [48]. Therefore, supplementing nutritional vitamin D might play an adjunctive role in treating and preventing infections in dialysis patients.

Our study has several limitations. Firstly, we cannot apply our findings or draw definitive conclusions in the general HD population due to relatively small sample size, however the results were indeed promising and clinically important. We used total calcium concentrations rather than ionized calcium concentrations, which could have some effects on vitamin D levels. Furthermore, we excluded patients with severe malnutrition, and inflammatory or infectious disorders who might have benefited the most from the intervention due to vitamin D deficiency. However, our cohort had significantly low vitamin D levels in both arms. Next, we did not determine the morbidity or mortality benefits of combination therapy. Although we performed some questionnaires on acute respiratory tract infections, the results were inconsistent and non-significant due to shorter follow-up duration. The optimal serum 25(OH)D_3_ target level in dialysis patients remains unknown, however, we supposed some additive PTH lowering effects with 25(OH)D_3_ ≥ 30 ng/dL. The beneficial role of cholecalciferol in chronic kidney disease-mineral bone disorder (CKD-MBD) requires a multifaceted long-term approach and needs further study in larger clinical trials. The results from our study showed that nutritional vitamin D compounds synergizes with active vitamin D compounds in lowering PTH and should be more broadly considered among HD patients with SHPT.

In conclusion, our findings provide in vivo evidence that 25(OH)D_3_ repletion with native vitamin D acts additively with active vitamin D analogs for controlling SHPT among HD patients. We further confirm the positive relationship between 25(OH)D_3_ and cathelicidin levels in these patients. Future large-scale clinical trials are warranted to justify the incorporation of 25(OH)D_3_ repletion into SHPT treatment recommendations in dialysis patients.

## Figures and Tables

**Table 1:** Demographic characteristics, diagnosis of renal disease and baseline data in the two groups under study (*n* = 30 each).

Characteristics	Paricalcitol + Placebo (*n* = 30)	Paricalcitol + Cholecalciferol (*n* = 30)	*p* Value
Age, mean ± SD (years)	65.2 ± 12.4	64.8 ± 13.2	0.904
Male, *n* (%)	16 (53.3%)	14 (46.7%)	0.941
BMI (kg/m^2^)	22.92 ± 3.48	23.37 ± 4.02	0.65
HD vintage (months)	49.85 ± 35.33	54.03 ± 26.67	0.52
GN, *n* (%)	5 (16.7%)	6 (20%)	0.959
DM, *n* (%)	13 (43.3%)	12 (40%)	0.944
HTN, *n* (%)	4 (13.3%)	3 (10%)	0.99
Others, *n* (%)	8 (26.6%)	9 (30%)	0.952
Prior calcitriol usage, *n* (%)	14 (46.7%)	15 (50%)	0.937
iPTH (pg/mL)	681.9 ± 173.3	689.0 ± 180.1	0.877
25(OH)D_3_ (ng/mL)	19.53 ± 8.2	19.6 ± 7.3	0.972
hCAP-18 (ng/mL)	24.05 ± 7.99	22.25 ± 6.71	0.349
Albumin (g/dL)	3.95 ± 0.34	3.82 ± 0.37	0.162
Alkaline phosphatase (U/L)	121.2 ± 48.4	128.8 ± 52.4	0.562
cCa (mg/dL)	9.19 ± 0.63	9.22 ± 0.51	0.840
P (mg/dL)	5.02 ± 0.78	5.14 ± 0.74	0.543

Data are expressed as the mean ± SD, *n* (%), or median (interquartile range). SD: standard deviation; BMI, Body Mass Index; HD, Hemodialysis; GN, primary glomerulonephritis; DM, diabetes mellitus; HTN, hypertension; Others, vascular or ischemic nephropathy and tubulointerstitial nephritis; iPTH, parathyroid hormone; 25(OH)D_3_, 25-Hydroxyvitamin D; hCAP-18, serum human cathelicidin, cCa, albumin-corrected calcium; P, phosphorus.

**Table 2:** Serum biochemistry parameters changes after 16 weeks of treatment.

Parameters	Paricalcitol + Placebo (Control)			Paricalcitol + Cholecalciferol (Study)		
	Before	After	*p* Value	Before	After	*p* Value
iPTH (pg/mL)	681.9 ± 173.3	299.37 ± 74.62	<0.05	689.0 ± 180.08	262.9 ± 57.14	<0.05
25(OH)D_3_ (ng/mL)	19.53 ± 8.2	19.63 ± 7.56	0.96	19.63 ± 7.26	30.4 ± 7.7	<0.05
hCAP-18 (ng/mL)	24.05 ± 7.99	26.59 ± 65.68	0.075	22.25 ± 6.71	82.13 ± 68.67	<0.01
cCa (mg/dL)	9.19 ± 0.63	9.28 ± 0.72	0.61	9.22 ± 0.51	9.18 ± 0.62	0.46
P (mg/dL)	5.02 ± 0.78	5.08 ± 0.82	0.77	5.14 ± 0.74	5.07 ± 0.62	0.69

iPTH, parathyroid hormone; 25(OH)D_3_, 25-Hydroxyvitamin D; hCAP-18, serum human cathelicidin, cCa, albumin-corrected calcium; P, phosphorus.

**Table 3:** Number of study patients achieving primary and secondary outcomes between paricalcitol and dual vitamin D treatment group.

Primary Outcome	4th Week	8th Week	12th Week	16th Week
iPTH ≤ 300 pg/mL	*n*/30 (%)	*n*/30 (%)	*n*/30 (%)	*n*/30 (%)
Paricalcitol	2/30 (6.7)	7/30 (23)	12/30 (40)	15/30 (50)
Paricalcitol + Cholecalciferol	2/30 (6.7)	7/30 (23)	18/30 (60)	23/30 (76.7)
	*p* = 1.000	*p* = 1.000	*p* = 0.121	*p* = 0.032
**Secondaty Outcome**	**4th Week**	**8th Week**	**12th Week**	**16th Week**
25(OH)D_3_ ≥ 30 ng/dL				
Paricalcitol	6/30 (20)	2/30 (6.7)		3/30 (10)
Paricalcitol + Cholecalciferol	4/30 (13.3)	15/30 (50)		18/30 (60)
	*p* = 0.488	*p* = 0.001		*p* = 0.001
Double of hCAP-18				
Paricalcitol				2/30 (6.7)
Paricalcitol + Cholecalciferol				12/30 (40)
				*p* = 0.006
